# Investigating the role of phonons in the phase stability of uranium-based Laves phases†‡

**DOI:** 10.1039/d3ra00498h

**Published:** 2023-03-15

**Authors:** Erik Nykwest, Ashley E. Shields, Z. E. Brubaker, J. L. Niedziela, S. B. Isbill, Andrew Miskowiec

**Affiliations:** a Nuclear Nonproliferation Division, Oak Ridge National Laboratory USA nykwestec@ornl.gov shieldsae@ornl.gov

## Abstract

Laves phase alloys possess unique thermal and electrical conduction properties, yet the factors governing phase stability in these systems remain an open question. The influence of phonons in particular has been broadly overlooked. Here, we investigate the UCo_2*x*_Ni_2(1−*x*)_ chemical space using density functional theory, which offers a unique opportunity to explore the factors influencing Laves phase stability as all three primary Laves phases (C14, C15, C36) can be stabilized by changing the ratio of Co to Ni. Calculations of the thermodynamic and dynamical stability of pure UCo_2_ and UNi_2_ in each of three primary Laves phases confirm the stability of experimentally known Laves phases for UNi_2_ and UCo_2_. A decrease in bonding strength is identified in UNi_2_ compared to UCo_2_, aligned with redshifts observed in the UNi_2_ phonon density of states and a decoupling of the U and Ni vibrational modes. Phonon calculations of C14 UCo_2_ reveal dynamical instabilities. Efforts to remove the unstable mode at the *Γ* point in UCo_2_*via* atomic displacements break the symmetry of the C14 phase, revealing a lower energy *P*2/*c* structure. Vibrational contributions to the free energy were calculated and did not change the thermodynamically stable Laves phase below 1000 K. The temperature-dependent free energies of single phase UCo_2_ and UNi_2_ were used to interpolate the relative stability of ternary UCo_2*x*_Ni_2(1−*x*)_ in each of the three Laves phases at varying temperatures and stoichiometries. The ternary C36 phase is only predicted to be thermodynamically stable over a narrow stoichiometric range below 600 K.

## Abstract 2

DFT investigations of the thermodynamic stability of UCo_2_ and UNi_2_ for three Laves phase structure types were extrapolated to UCo_2*x*_Ni_2(1−*x*)_.

## Graphical abstract

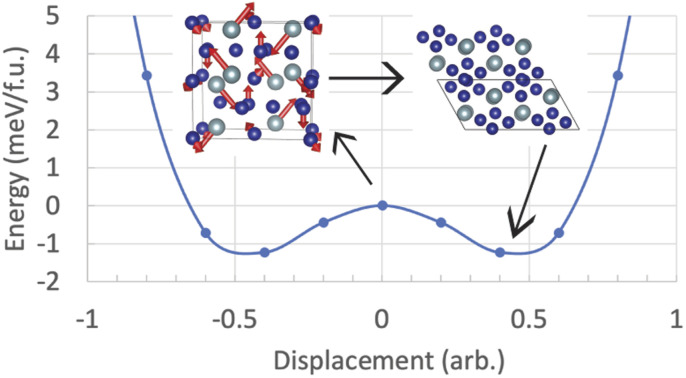

## Introduction

Because of the United States' commitment to reduce net greenhouse gas emissions by 2030,^1^ there is increased interest in using uranium alloys as fuel for nuclear reactors. Metallic fuels naturally transport heat more easily than ceramic fuels and can be shaped to allow more water to flow across the fuel rod's surface, further increasing heat transfer and electricity generation. Higher surface areas also promise an increased margin of safety to the reactor core.^2,3^ With the increasing interest of using uranium alloys in the nuclear fuel cycle, understanding the influence of alloy formation in these materials is critical.

**Fig. 1:**
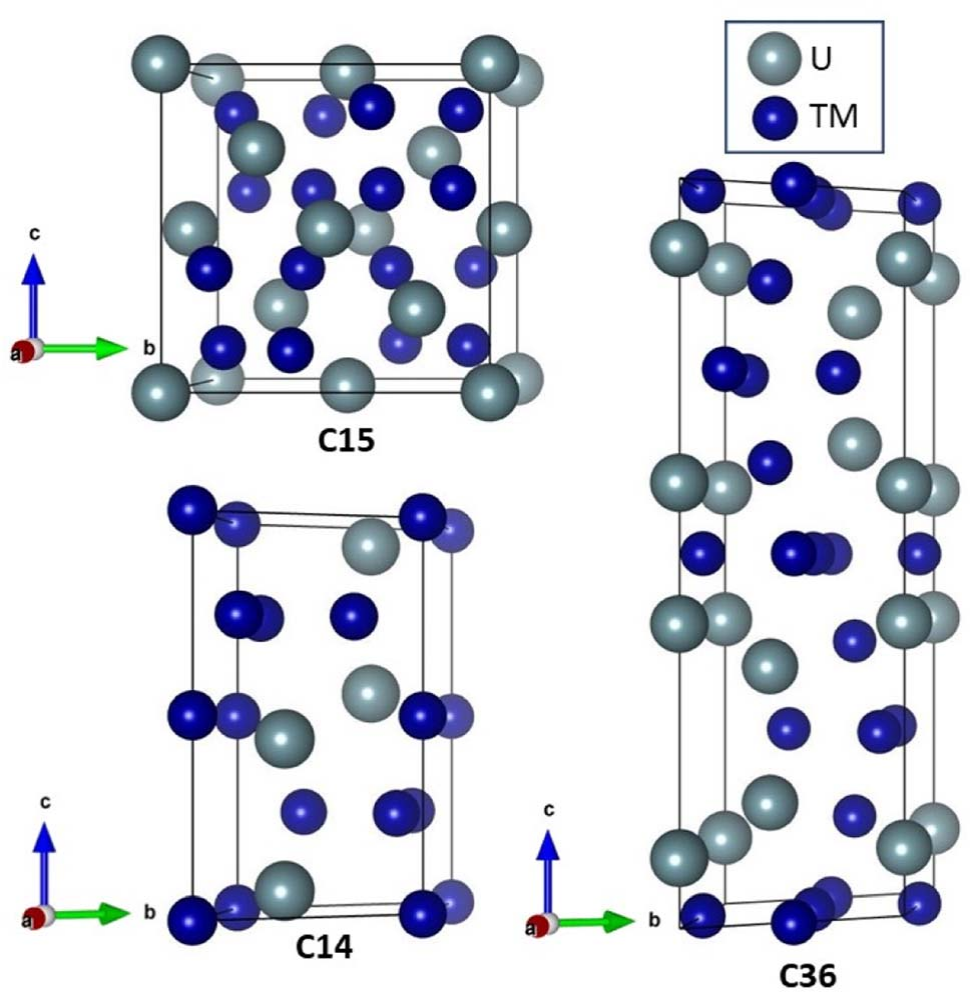
Unit cells for the three main Laves phase structure types, (bottom left) C14, (top left) C15, and (right) C36. The larger uranium atom sits on the A sites, and the smaller transition metals sit on the B sites.

When uranium combines with a steel constituent (*e.g.*, transition metal elements) a Laves phase is a common by-product. Laves phases account for more than 1400 materials and are formed by elements in all regions of the periodic table.^4,5^ Laves phases form three crystal structures, shown in Fig. 1, denoted as C14 (hexagonal, *P*6_3_/*mmc*), C15 (cubic, *Fd*3̄*m*), and C36 (hexagonal, *P*6_3_/*mmc*), and only differ by the stacking arrangement of the individual layers.^5^ Longer, more complex stacking patterns are possible but uncommon.^6,7^ Laves phases possess AB_2_ stoichiometry and exhibit novel thermophysical properties because of their interconnected network of tetrahedral structures, which facilitates high electrical and thermal conductivity. Owing to their high melting point, controlled precipitation of Laves phases can strengthen steels and superalloys.^5,8^ Laves phases are also the most frequently found intermetallic phase in high-entropy alloys.^9^ A significant amount of experimental, computational, and theoretical work has been performed on Laves phases; however, the factors dictating their structural stability remain an open question.^10,11^ Part of the difficulty stems from the vast number of Laves phases. This difficulty is exacerbated by the insufficient reliability of many phase diagrams due to experimental difficulties associated with the necessary measurements.^11^ Nonetheless, the stability of some Laves phase structures appear to be correlated with the valence electron configuration,^12–14^ relative atomic sizes,^12,14^ and electronegativity.^15–20^ Unfortunately, none of the developed models have been able to provide a general description of the relative stability between the different Laves phase structures across the large class of Laves phase-forming compounds.^10,21^ Surprisingly, the role of phonon interactions has been widely overlooked, despite studies linking phonon instabilities to structural transitions in other materials.^22–25^

In this study we attempt to gain insight into the factors affecting the structural preferences of uranium-based Laves phase systems by calculating and comparing the free energy, phonon density of states (phDOS), and electronic density of states (eDOS) of pure UCo_2_ and UNi_2_ in each of the three main Laves phases.^11^ The ternary U–Co–Ni system offers a promising subset of structures to investigate structural stability. To our knowledge, UNi_2_ is the only Ni-containing binary compound that forms in the C14 structure,^26^ and UCo_2_ forms the C15 structure.^27^ Experimental measurements of UCo_2*x*_Ni_2(1−*x*)_ claim that at small concentrations of Co (*x* < 0.12) the system stabilizes in the hexagonal C14 phase, at moderate concentrations (0.40 < *x* < 1.0) the cubic C15 phase is dominant, and in between these concentrations a mixture of phases is found, including the C36 phase.^28,29^ Currently there are only six different U-bearing alloys (U–Al, U–Al–Co, U–Ir–Al, U–Al–Ni, U–Os–Al, U–Co–Ni) with stable C36 phases reported in the Inorganic Crystal Structure Database,^29–34^ three of which (U–Al–Co, U–Os–Al, U–Ir–Al) are the subject of some dispute as to whether they truly form a stable C36 structure or not.^35–37^ The fourth structure (UAl_2_) is only stable at high pressures, and the fifth (U–Al–Ni) has the U atom on the B site instead of the A site. Therefore, ternary U–Co–Ni may be the only uranium-based C36 Laves phase that is stable under atmospheric conditions.

Although recent research has been performed on uranium-transition metal Laves phases,^38,39^ most was conducted before the 1990s and heavily focused on the magnetic properties and not phase stability. Most of this past research was experimental,^40–48^ with few theoretical studies.^47,49,50^ Because of computational limitations, past investigations were restricted to a small number of atoms, rather than the complete unit cell. The combination of improvements in high-performance computing systems and the underlying theory, particularly development of the local density and generalized gradient approximations in density functional theory (DFT), now allow more complete investigation of the electronic structure of complex materials, including the symmetry-breaking displaced structures required for calculating phonon band structures. To specifically address the role of phonon interactions in phase stability, we have computationally investigated pure UCo_2_ and UNi_2_ in each of three Laves phases using DFT.

## Methodology

To model the C15 phase, the standard cubic unit cell was used. To reduce computational complexity, the hexagonal C14 and C36 phases were transformed to equivalent orthorhombic unit cells using the transform in eqn (1).1
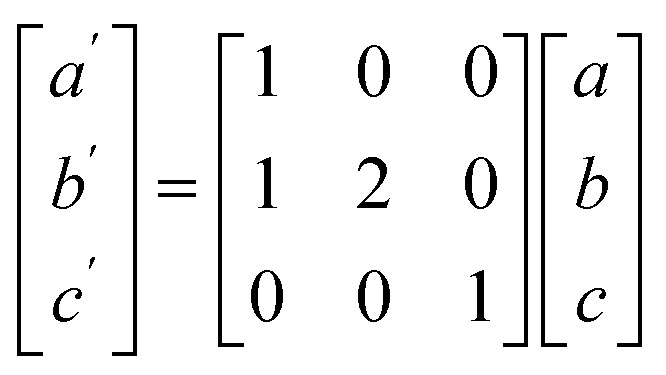


In terms of representing the crystal structure, the larger orthorhombic unit cell is equivalent to the traditional hexagonal unit cell but contains more atoms, similar to using a super cell, and does not change the underlying symmetry. Herein, the C14 and C36 will continue to be referred to as hexagonal phases.

Spin-polarized DFT calculations were performed in the Vienna *ab initio* simulation package (VASP) 5.4.4 ^51^ using the generalized gradient approximation PBEsol^52^ exchange–correlation functional, projector augmented wave pseudopotentials,^53^ periodic boundary conditions, and *Γ*-centered Monkhorst–Pack *k* point meshes.^54^ To achieve energy predictions converged to <1 meV per atom, a 3 × 3 × 5 *k* point mesh was used for the C14 structure, a 7 × 7 × 7 mesh for the C15 structure, and a 5 × 3 × 2 mesh for the C36 structure. All calculations were performed with a plane wave cutoff of 400 eV.

Structural relaxations were performed using the conjugant gradient method while allowing cell volume, shape, and ionic positions to relax simultaneously until atomic forces were less than 1 meV Å^−1^ with a 0.1 eV Methfessel–Paxton smearing^55^ of first order applied to the electron occupations at the Fermi level. Single point electronic density of states calculations (eDOS) utilized the tetrahedron method with Blöchl corrections.^56^ To determine how each element contributes to the eDOS, the calculated wavefunctions were projected onto spherical harmonics centered on each atom in the simulation cell. Magnetic moments were initialized on all atoms using a ferromagnetic ordering with a magnitude of 1 bohr-magneton, which is the default initialization condition in VASP.

The phonon band structure was calculated *via* the finite displacement method, where Phonopy^57^ was used to generate symmetrically inequivalent displacements of atoms in the unit cells, and VASP was used to calculate the internal energies of the perturbed systems. Postprocessing in Phonopy determined the force constants, phonon band structure, atom-projected density of states, heat capacity, entropy, and entropic contributions to the free energy for the system. The Python module Sumo^58^ was used to determine the symmetry group of the relaxed structures using a tolerance of 0.01 Å and to generate *q*-point paths through reciprocal space consistent with Bradley and Cracknell.^59^

From calculated phonon properties, we can extract thermal properties for these Laves phases. The thermodynamically stable phase of a system minimizes the relevant energy function for that system; for solids this is typically the Helmholtz free energy (*F*):

2 *F* = *U*_s_ + *U*_0_ − *TS*,

where *U*_s_ is the structural internal energy, *U*_0_ is the zero-point energy, *T* is the temperature, and *S* is the entropy of the system. To obtain internal structural energies, both UCo_2_ and UNi_2_ were modeled using DFT in all three Laves phases, C14, C15, and C36. The zero-point energy and entropy are calculated from the phonon band structure within the harmonic approximation.

The free energy of the mixed UCo_2*x*_Ni_2(1−*x*)_ system can be written as

3 *F*_*ϕ*_(*x*) = *xF*_*ϕ*_(UCo_2_) + (1 − *x*)*F*_*ϕ*_(UNi_2_) + *δF*_m_(*x*,*ϕ*),

where *F*_*ϕ*_(*x*) is the total free energy of UCo_2*x*_Ni_2(1−*x*)_ in the *ϕ* phase, with *ϕ* ∈ {C14, C15, C36}, and *F*_*ϕ*_(…) is the free energy of pure UCo_2_ or UNi_2_ in the *ϕ* phase, and *δF*_m_(*x*,*ϕ*) is the energy of mixing. Alternatively, the mixed system may segregate into pure UCo_2_ and UNi_2_. The free energy of a segregated system (*F*_seg_) is given by

4 *F*_seg_ = *xF*_min_(UCo_2_) + (1 − *x*)*F*_min_(UNi_2_) + *δF*_gb_(*x*,*ϕ*_1_,*ϕ*_2_),

where *F*_min_(…) is the free energy of pure UCo_2_ or UNi_2_ in its lowest energy phase (C15 for UCo_2_ and C14 for UNi_2_), and *δF*_gb_(*x*,*ϕ*_1_,*ϕ*_2_) is the grain boundary energy between grains of phase *ϕ*_1_ and *ϕ*_2_. The grain boundary energy includes both the change in energy of existing grain boundaries due to segregation as well as energy of creating new grain boundaries between dissimilar phases (*e.g.*, for a pure single crystal of C36 to segregate into two grains, one of C14 and one of C15, one or more grain boundaries must be formed between the two phases). In this work *δF*_m_ and *δF*_gb_ are not calculated directly, as any calculated interface energy would be configuration specific. Instead, the effects of *δF*_m_ and *δF*_gb_ on ternary phase stability is investigated through the adjustable parameter *δF*.

5 *δF* ≡ (*δF*_gb_(*x*) − *δF*_m_(*x*))

## Results and discussion

### Relaxed structures and unstable phonons in UCo_2_–C14

**Fig. 2:**
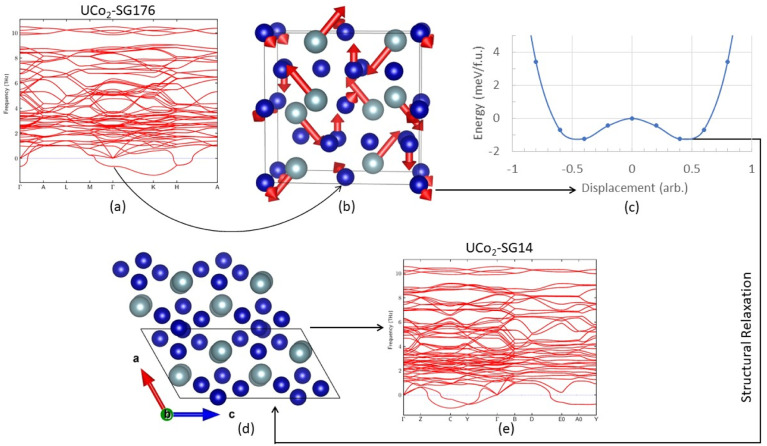
(a) The phonon band structure for UCo_2_ in the SG176 structure (relaxed C14). Unstable phonon modes (imaginary frequencies) are plotted as negative frequencies, showing an instability at the *Γ* point. (b) Atomic displacement vectors associated with the unstable vibrational mode at the *Γ* point in (a) are indicated by red arrows. (c) The energy surface associated with displacing the atoms of UCo_2_-SG176 along the vibrational direction of the unstable phonon mode at the *Γ* point, shown in (b). The interpolating line is for visual purposes only. The *x*-axis shows the approximate displacement of U atoms in angstrom (each atom moves a different amount according to the eigenvector). (d) A 2 × 1 × 1 rendering of UCo_2_-SG14 in a standardized monoclinic unit cell (outlined in black). The monoclinic unit cell is similar in shape and length to a 1 × 2 × 1 super cell of the original hexagonal C14 unit cell. (e) The phonon band structure for UCo_2_ in the relaxed SG14 structure, showing the structure modification stabilizes the *Γ* point, but still possesses unstable modes.

Before discussing results across structure classes, we make an observation about a dynamic instability identified in the UCo_2_ investigations. During atomic relaxation, the UCo_2_–C14 structure underwent spontaneous symmetry breaking, reducing the space group from 194 to 176 in the DFT optimized structure. From here on the relaxed UCo_2_–C14 structure will be referred to as SG176. Examination of the phonon band structure of UCo_2_-SG176, Fig. 2a, revealed vibrational modes with imaginary frequencies, indicating this structure is dynamically unstable. To determine if these unstable modes were an artifact of our methodology, we applied several different techniques such as using density functional perturbation theory instead of finite differences to calculate the phonon modes and performing phonon calculations on a super cell of the relaxed structure, neither of which successfully eliminated all imaginary frequencies. The resilience of these imaginary frequencies suggests they are not simply computational artifacts but real dynamic instabilities.

To probe this hypothesis, the atoms of the relaxed structure were displaced along the eigenvectors of the unstable vibrational mode. The eigenvector components associated with the unstable mode at the *Γ* point are shown in Fig. 2b. To remove the unstable mode at the *Γ* point from the band structure, the atoms of UCo_2_-SG176 were displaced along their associated vibrational paths (indicated by red arrows), and single point energy calculations were performed at various displacement amplitudes. The energy surface associated with the displacement magnitude is shown in Fig. 2c. These calculations reveal there is no energy barrier between the SG176 structure and a lower energy, lower symmetry structure. This lower energy intermediate structure was relaxed using VASP, and the relaxed structure is referenced herein as SG14, as the symmetry belongs to the monoclinic space group number 14. The SG14 structure is shown in Fig. 2e and is 2.7 meV f.u.^−1^ lower in energy than SG176. Note that the eigenvector displacement successfully removed the unstable optical mode from the SG14 band structure at the *Γ* point (Fig. 2d), but imaginary frequency modes are present off the *Γ* point. The eigenvector displacement method previously described was also applied to the imaginary frequency modes at C, Y, E0, and A0, but the corresponding energy surfaces were concave parabolas and did not reveal any nearby minima. Nevertheless, this analysis shows that, at this level of theory, UCo_2_ is dynamically unstable in the C14 structure as there is no energy barrier to overcome during the C14 to SG14 transition.

The lattice constants and internal energy of all systems investigated in this work, including the newly identified UCo_2_-SG14, are listed in Table 1. The structural parameters for the known ground states of UCo_2_ and UNi_2_ are in good agreement with experiment and the energetic predictions correctly identify the experimentally known stable structures^28^ (C15 for UCo_2_ and C14 for UNi_2_) as having the lowest internal energy. Note, the most stable phase for UCo_2_ is the least stable phase for UNi_2_, and *vice versa*. The supposed appearance of the C36 phase in the ternary system may be an attempt to balance the energetic structural preferences of Co and Ni, as the calculated internal energy of the C36 phase sits between the C14/SG14 and C15 phases for both the Co and Ni systems.^28^

The structural properties, space groups, internal energies, and spin magnetic moments of the relaxed (a) UCo_2_ and (b) UNi_2_ Laves phase structures are shown below. The experimental ground state structure at ambient temperatures and pressures for each system has been marked with an asterisk. Each reconstructed unit cell contained eight formula units, except for UNi_2_–C14, which contained four formula units. Cells marked with a dash are either known by symmetry or were unreported by the authors

**Table:** 

(a) UCo_2_	SG14	C15*	C36	Exp*^27^
Internal energy (eV f.u.^−1^)	−28.2614	−28.4085	−28.3286	—
Spin moment (*μ*_B_ f.u.^−1^)	0.00	0.59	0.54	—
Space group number	14	227	194	227
*a* (Å)	4.743	6.805	4.789	6.992
*b* (Å)	8.04933	—	—	—
*c* (Å)	9.520	—	15.878	—
*α* (°)	90	90	90	—
*β* (°)	119.41	—	90	—
*γ* (°)	90	—	120	—

**Table:** 

(b) UNi_2_	C14*	C15	C36	Exp*^26^
Internal energy (eV f.u.^−1^)	−25.1095	−25.0249	−25.0819	—
Spin moment (*μ*_B_ f.u.^−1^)	1.17	1.20	1.20	—
Space group number	194	227	194	194
*a* (Å)	4.830	6.901	4.847	4.9701(4)
*b* (Å)	—	—	—	—
*c* (Å)	8.125	—	16.149	8.2527(8)
*α* (°)	90	90	90	—
*β* (°)	90	—	90	—
*γ* (°)	120	—	120	—

### Electronic density of states

**Fig. 3:**
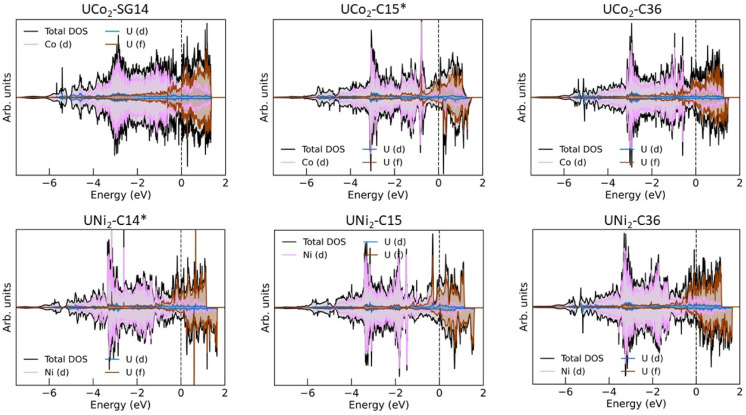
The electronic density of states after structural relaxation for UCo_2_ (UNi_2_) is shown in the top row (bottom row) for the SG14 (C14) structure on the left, C15 structure in the middle, and C36 structure on the right. The total density of states is indicated by a black line and grey infill, the electronic states attributed to U atom d-states are shown in blue, U f-states are shown in brown, and the d-states attributed to the transition metals, Co or Ni, are pink. The Fermi level has been shifted to 0 eV and is indicated by a vertical black dashed line. The experimentally stable structures of UCo_2_ and UNi_2_ are marked with asterisks.

Much work has already been performed investigating the magnetic properties of uranium alloys,^40,42,49,60–62^ and a detailed study of the magnetic properties of UCo_2_ and UNi_2_ are beyond the scope of this work. However, some comments about the eDOS can still be made. Fig. 3 shows the eDOS for UCo_2_ and UNi_2_ decomposed into their atomic and orbital contributions.

The majority of the conduction band is formed from the transition metal (TM) 3d states and the uranium 5f states, consistent with past calculations.^49,60^ Broadly, the eDOS can be understood as having a narrow U 5f band above the Fermi level and a broad TM 3d band below the Fermi level, with significant hybridization between these orbitals within 1 eV of the Fermi level. A nontrivial number of states display a U 6d-like character that spans the entire width of the valence band. Not shown is the band edge near −6 eV, which primarily comprises hybridized TM 4s and U 6d states.

There is little to no spin splitting (exchange splitting) of the TM 3d orbitals, evident from the near-mirror symmetry between the spin up (majority spin channel) and spin down (minority spin channel) states. This lack of spin splitting also holds for the U 5f states of UCo_2_-SG14. For each of the other structures, we observe exchange splitting of the U 5f states causing the spin down states to shift higher in energy compared to their corresponding spin up states. This spin splitting causes an asymmetric occupation of the eDOS creating a polarized ground state and a net spin magnetic moment attributable to the uranium 5f electrons. This outcome is consistent with previous DFT studies and neutron diffraction measurements that found UNi_2_–C14 to exhibit itinerant ferromagnetism,^40,60^ and polarized neutron diffraction studies of UNi_2_–C14 found it to have a low Curie temperature (*T*_C_) of 21 K.^40^ The net spin magnetic moment for each system is listed in Table 1. Based on the eDOS and their net spin magnetic moment, it would not be surprising if all the UNi_2_ Laves phases displayed itinerant ferromagnetism.

UCo_2_–C15 is thought to be paramagnetic.^49,63^ Our calculations predict a spin-polarized ground state with a spin magnetic moment of 0.59 *μ*_B_ per uranium atom. Examining the eDOS for UCo_2_–C15, we see there is an unpaired spin down peak in the eDOS just above the Fermi level near 0.2 eV. It is possible that, near 0 K, UCo_2_–C15 displays magnetic ordering, but at finite temperatures (*i.e.*, above the *T*_C_) this spin splitting is reduced or overcome by thermal fluctuations. This conclusion is consistent with past experimental magnetic a.c. susceptibility studies that found UCo_2_ to have a *T*_C_ of about 7 K and concluded that magnetic clusters exist in UCo_2_.^63^ Another possibility is that UCo_2_–C15 behaves like UFe_2_, and the orbital and spin magnetic moments from the U atoms cancel out, leading to paramagnetic behavior.^48,64–66^ As a reminder, the DFT calculations performed in this work do not account for the orbital angular momentum of the electrons. The spin magnetic moment and eDOS for UCo_2_–C36 is like that of UCo_2_–C15, and thus is also expected to be paramagnetic or have a low *T*_C_.

Some studies have linked the total number of electronic states at the Fermi level to the structural stability,^67–69^ with stability decreasing as the number of states increases. The total number of spin up and spin down states at the Fermi level (*N*_EF_) are given in Table 2; this relationship seems to hold for UNi_2_. However, UCo_2_ only partially agrees with this idea. Although the stable C15 phase has the lowest number of states at the Fermi level, the dynamically unstable and energetically unfavorable SG14 phase has the next lowest number of states.

**Table:** The number of electronic states, *N*_EF_, (states eV^−1^ f.u.^−1^) at the Fermi level in (a) UCo_2_ and (b) UNi_2_ for each of the relaxed structures. The experimentally stable structures of UCo_2_ and UNi_2_ have been marked with an asterisk

	*N*_EF_ (spin up)	*N*_EF_ (spin down)	Total
**(a) UCo**_**2**_			
SG14	2.51(13)	2.69(06)	5.20
C15*	2.37(30)	1.49(02)	3.86
C36	4.28(41)	1.48(09)	5.76

**(b) UNi**_**2**_			
C14*	2.12(11)	1.88(10)	4.00
C15	4.80(02)	1.99(05)	6.78
C36	3.16(23)	1.31(06)	4.48

### Phonon density of states

**Fig. 4:**
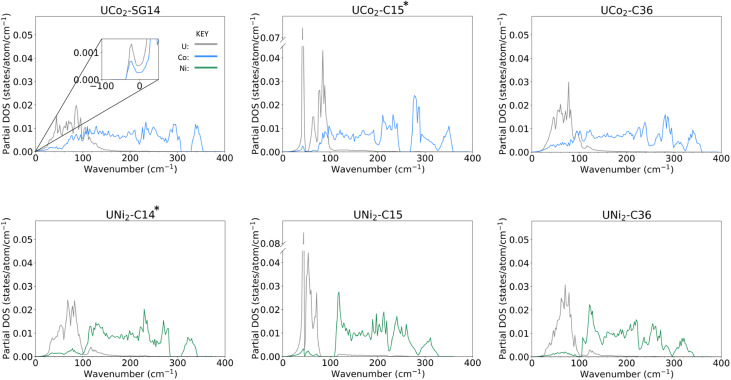
Atom decomposed phonon density of states for (top row) UCo_2_ and (bottom row) UNi_2_ in the (left column) SG14/C14, (middle column) C15, and (right column) C36 structures, after structural relaxation. Phonon DOS associated with Co atoms are shown in blue, Ni in green, and U in grey. The inset shows the UCo_2_-SG14 phDOS contains unstable phonon modes, represented by negative wavenumbers. The experimentally stable structures of UCo_2_ and UNi_2_ have been marked with an asterisk.

Examining the phonon density of states (phDOS) shown in Fig. 4, we see that the higher energy vibrational modes are primarily ascribed to Co/Ni vibrations, and the lower energy phonons are attributed to U atoms, which are consistent with the difference in masses of the contributing atoms. Fig. 4a shows the unstable phonon modes in the UCo_2_-SG14 phDOS as negative vibration frequencies, plotted in the inset. These unstable modes were discussed previously and shown in Fig. 2d. Note, none of the other Laves phases examined in this work exhibit unstable vibrational modes, including those that have not been explicitly experimentally determined. All phDOS terminate above ∼320 cm^−1^ for Ni phases and ∼350 cm^−1^ for Co phases.

The phDOS predicted for the experimentally observed UCo_2_–C15 phase shows a very sharp peak at low wave numbers, dominated by modes of uranium character. Fig. 4b shows a prominent uranium peak that goes off the scale, followed by an apparent quadruplet of states with nonzero density, which begin mixing with Co near 75 cm^−1^. The remaining uranium bands are largely positioned under 100 cm^−1^, with a very small contribution of the uranium atoms between 100 and 200 cm^−1^. In contrast, the UNi_2_–C15 phase shown in Fig. 4e also possess strong contributions of uranium character that go off the scale below 100 cm^−1^. Investigation of the band structure for both cases (see ESI Fig. 1‡) reveals these states in both the Co and Ni C15 structures to be nearly dispersionless, potentially suggesting a rattling-type mode may exist in these systems. In the C15 Ni system, the Ni dominated phonon bands are shifted to start above 100 cm^−1^, leaving a phonon band gap in the UNi_2_ phase between 70 and 110 cm^−1^. No low energy phonon gap exists in the C15 UCo_2_ phase.

For both C15 systems, the phonon spectra greater than 100 cm^−1^ are dominated by TM modes, as might be expected, though each display a long residual tail of low uranium involvement that terminates at ∼200 cm^−1^ for Co and ∼220 cm^−1^ for Ni, each termination of which is accompanied by a sharp drop in the contribution of the TM vibrations. For the C15 UCo_2_ phase, the experimentally stable configuration, there is a phonon band gap near 250 cm^−1^, and a phonon “pseudogap”, where the phonon contributions are nearly zero, near 320 cm^−1^. For the C15 UNi_2_ phase, there is a similar pseudogap near 290 cm^−1^.

Comparing the C14 analogues, in UCo_2_-SG14, Fig. 4a, there is a phonon band gap near 320 cm^−1^. This gap also exists in UNi_2_–C14, Fig. 4d, but it starts at ∼290 cm^−1^ extending to 310 cm^−1^. Similar to the C15 case for the Co system, the region of U–Co modes extends to higher energy compared to the U–Ni system, although the tail for continued uranium involvement extends to higher overall energy.

In the C36 phases, the low energy modes follow the same trend where the uranium motions are better separated from the TM contributions at low energy for the case of Ni compared to Co. There is the continued presence of a phonon pseudogap near 325 cm^−1^ for C36 UCo_2_ and near 290 cm^−1^ for C36 UNi_2_. Total phDOS for all phases are shown in ESI Fig. 2.‡

### Thermodynamic stability

**Fig. 5:**
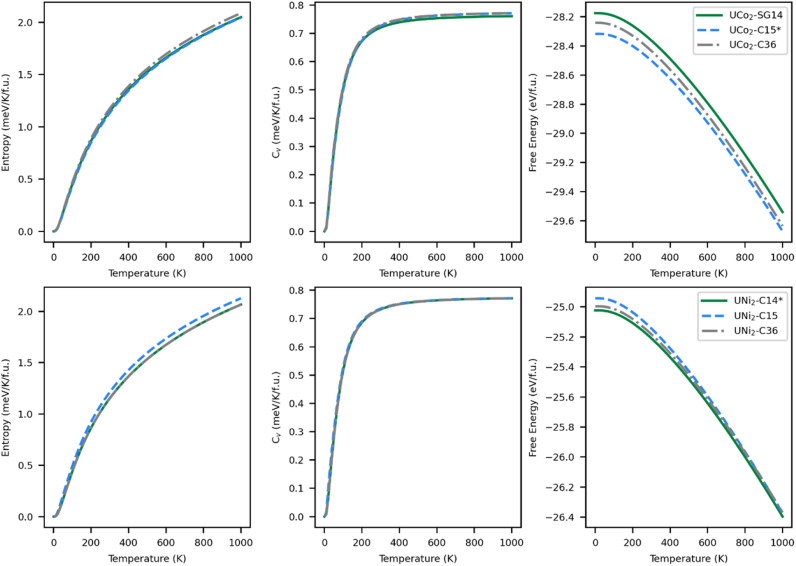
(Left) Entropy, (middle) heat capacity, and (right) free energy for (top) UCo_2_ and (bottom) UNi_2_ as a function of temperature. Lower free energy means the structure is more stable. Properties associated with the SG14/C14 phase are plotted as a green solid line, the C15 phase in blue dashes, and the C36 phase in grey dash-dots. The experimentally known structures of UCo_2_ and UNi_2_ have been marked with an asterisk.

The thermodynamically stable phase of a system minimizes the relevant energy function for that system; for solids this is typically the Helmholtz free energy (eqn (2)). Within the harmonic approximation, the entropic portion of the free energy may be estimated from the ground state vibrational properties. The accuracy of this method decreases with temperature but typically remains useful up to 1000 K.^70^ The entropy, specific heat at constant volume, and free energy are plotted in Fig. 5 as a function of temperature.

The entropy curves, and thus also the specific heats, of all the structures are similar. The specific heat saturates to about 0.77 meV K^−1^ f.u.^−1^ at high temperatures. Although entropy has a large effect on the free energy of the systems, generating a decrease in energy in excess of 1.4 eV f.u.^−1^, there is no change in the most stable structure as a function of temperature over the range modeled. Although the C14 phase remains the lowest energy phase for UNi_2_ and the C15 phase remains the lowest energy phase for UCo_2_, the energetic differences between the phases do get smaller with increasing temperature. For UCo_2_, the energetic ordering remains the same over the full range of 0–1000 K, with the C15 phase remaining the most stable phase and the SG14 phase the least stable. At 1000 K, UCo_2_–C15 is 37.1 meV f.u.^−1^ lower in energy than the C36 phase and 132.0 meV f.u.^−1^ lower than the SG14 phase. For UNi_2_, the energetic ordering does change, with the C15 phase becoming more stable than the C36 near 850 K. At 1000 K, UNi_2_–C14 is more stable than the C15 phase by just 22.7 meV f.u.^−1^ and 27.0 meV f.u.^−1^ lower in energy than the C36 phase. This energy difference is just less than 2% of the total free energy change due to entropic effects. Accordingly, it is worth investigating the phase stability of UNi_2_ Laves phases with higher accuracy methods or *via* experiment.

### Thermodynamic stability of ternary UCo_2*x*_Ni_2(1−*x*)_

The phase stability of ternary UCo_2*x*_Ni_2(1−*x*)_ may be estimated by comparing the free energy of a single phase ternary system in each of the three Laves phase, predicted with eqn (3), with the free energy of the segregated multiphase system *x*UCo_2_ + (1 − *x*)UNi_2_, given by eqn (4).

We first examine ternary phase stability without vibrational effects by setting the zero-point energy and temperature–entropy contributions to zero. If we assume *δF*_gb_ and *δF*_m_ are phase independent, it can be shown that for *δF* ≤ 0 eV f.u.^−1^ (*i.e.*, the energetic penalty for mixing is greater than or equal to the penalty for creating grain boundaries in eqn (5)), it is always energetically favorable for the system to segregate into pure UCo_2_–C15 and UNi_2_–C14. For 0 < *δF* < 42.3 meV f.u.^−1^, (*i.e.*, the penalty for creating grain boundaries is larger than the energy of mixing), it is possible to find ratios of Co to Ni that stabilize ternary C14–SG14 and C15, but not C36. As *δF* increases from 42.3 meV f.u.^−1^ the ratio where it is possible to stabilize the C36 increases until 49.3 meV f.u.^−1^, above which there are no further changes to the phase stability regions and the ternary system is not predicted to segregate into pure phases. Using this maximum difference in energetic penalty, the ternary C14–SG14 phase is stable for 0 < *x* < 0.28, the C36 phase is stable for 0.28 < *x* < 0.42, and the C15 phase is stable for 0.42 < *x* < 1.0. The C36 to C15 boundary at *x* = 0.42 is in good agreement with experiments by Zeleny *et al.*^29^ who found a boundary near *x* = 0.38. The C14 to C36 boundary of *x* = 0.12 measured by Zeleny *et al.*, however, is much lower than we predict using structural energy alone.

**Fig. 6:**
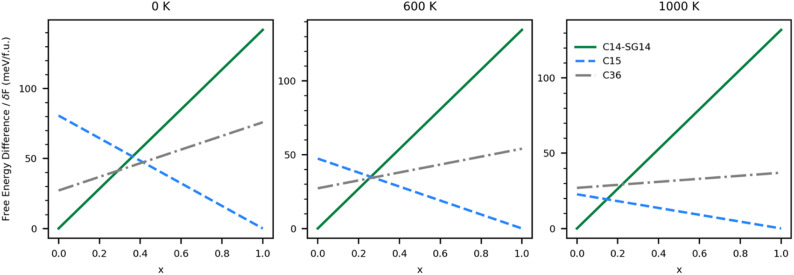
The estimated free energy differences, including vibrational contributions, for ternary UCo_2*x*_Ni_2(1−*x*)_ in the C14–SG14 (solid green), C15 (blue dashes), and C36 (grey dash dot) phases calculated relative to segregated *x*UCo_2_ + (1 − *x*)UNi_2_, assuming the energetic penalty of segregation (*δF*) of zero, is shown for (left) 0 K, (middle) 600 K, and (right) 1000 K. The minimum energetic penalty (*δF*) required to stabilize the lowest energy single phase at any given value of *x* is equal to this relative free energy.

This same analysis can be applied to the system with vibrational effects by using the free energies shown in Fig. 5; however, the predictions become a function of temperature. Fig. 6 shows the free energy differences of each ternary single-phase system relative to a segregated system at three different temperatures. In the absence of an energetic penalty to segregation (*e.g.*, grain boundary energy), the ternary system is not stable at any temperature, indicated by all of the relative free energies being greater than zero. The minimum energetic penalty predicted to stabilize the lowest energy single phase at any given value of *x* is shown as the free energy difference relative to the segregated system.

As expected, the 0 K system is predicted to behave similarly to the vibration-free system. As *δF* grows, it becomes possible to stabilize the C14 phase at low Co (high Ni) concentrations and C15 at high Co (low Ni) concentrations, but at intermediate values of *x*, the system thermodynamically favors segregation. As *δF* increases from 41 to 47 meV f.u.^−1^, it becomes possible to stabilize the C36 phase. As temperature increases, the *x* range over which the C36 phase is thermodynamically stable shrinks until around 600 K. Above 600 K our calculations predict the C36 phase is no longer thermodynamically stable at any value of *x*, and the C14 phase yields thermodynamic stability to the C15 phase directly when *x* > 0.28. The values of *δF* over which the stability regions change are listed in Table 3. Energetic penalties larger than those listed in the table will also stabilize the ternary single phases, but the stability regions will remain unchanged.

**Table:** The minimum energetic penalty (*δF*) in meV f.u.^−1^ required to stabilize ternary UCo_2*x*_Ni_2(1−*x*)_ in each of the phases and temperatures investigated. If a value is omitted, that means the phase is thermodynamically unstable at that temperature

	C14	C15	C36
0 K	0–41	41–47	0–47
600 K	0–34	0–34	—
1000 K	0–19	0–19	—

Assuming the *δF* is large enough to stabilize the C36 phase, we can again determine what ranges of *x* stabilize each of the three Laves phases investigated, although experimentally Zeleny *et al.*^29^ report that the single phase regions are separated by narrow two phase domains. These ranges are shown in Table 4. At 0 K, the C36 phase is predicted to be stable in the stoichiometric region between the C14 and C15 phases. Further experimental and computational efforts may find C36 is an intermediate phase in the transition between C14 and C15 in the ternary system.

**Table:** The phase stability regions of ternary UCo_2*x*_Ni_2(1−*x*)_ in each of the phases and temperatures investigated, assuming the system does not segregate into *x*UCo_2_ + (1 − *x*)UNi_2_

	C14	C15	C36
0 K	0 < *x* < 0.29	0.41 < *x* < 1	0.29 < *x* < 0.41
600 K	0 < *x* < 0.28	0.28 < *x* < 1	—
1000 K	0 < *x* < 0.17	0.17 < *x* < 1	—

As temperature increases, changes in the phase diagram are driven primarily by changes in the relative free energy of UNi_2_ Laves phases. As can be seen from Fig. 5, the free energy difference between the C15 phase of UNi_2_ and the other phases decreases with temperature, with the C15 phase becoming more stable than the C36 phase above 850 K. This increase in relative stability of the UNi_2_ in the C15 phase flattens the ternary C15 energy curve in Fig. 6, creating large changes in the C15 phase stability region as a function of temperature. This effect both increases the uncertainty in any reported C15 phase stability interval due to its high sensitivity to temperature and could also explain the discrepancy between our predicted C15 stability region and experiment. Using the free energies calculated at 270 K, we find a C36 to C15 transition at *x* = 0.37 in excellent agreement with Zeleny *et al.*,^29^ who report the transition at *x* = 0.38. However, at 270 K we find the C14 to C36 transition occurs at *x* = 0.29, which is barely different from the vibration free transition and still disagrees with the 0.12 < *x* < 0.17 range reported in their manuscript.

## Conclusions

Currently, there is not one unifying theory for predicting relative phase stability that works across the large class of Laves phase-forming compounds and the effects of phonon contributions to the relative stability have largely been overlooked. Here, DFT was used to predict the structural, vibrational, energetic, magnetic, and electronic properties of UCo_2_ and UNi_2_ in each of the three main Laves phases. DFT confirmed the experimentally determined stable phases for each chemistry are the lowest energy phases, C14 for UNi_2_ and C15 for UCo_2_. DFT calculations also predict stable phonon structures for all but the UCo_2_ SG14 phase.

The interatomic distances of UNi_2_ were typically larger than those seen in the corresponding UCo_2_ phases, suggestive of a decrease in bond strength. The proposed decrease in bond strength also aligned with observations of the phDOS, in which the UNi_2_ phDOS was redshifted compared to UCo_2_. In addition, the phDOS displayed a decoupling of the U and Ni vibrational modes. We have identified a modification to UCo_2_–C14, which we refer to as SG14, that lowers the internal energy of the structure and removes the unstable phonon mode at the *Γ* point. Other imaginary modes exist off *Γ*, but the SG14 structure can serve as a starting point for future research. There is no energetic barrier between the C14 structure and the SG14 structure.

Although the relative free energies change, vibrational contributions did not change the thermodynamically stable Laves phase below 1000 K. There may be a phase transition above 1000 K as the harmonic approximation breaks down at elevated temperatures, and the predicted energy differences decrease with increasing temperature, especially for UNi_2_. Accordingly, it is worth investigating the phase stability of UNi_2_ Laves phases with higher accuracy methods or *via* experiment.

Finally, the ternary C36 phase was found to only be thermodynamically stable below 600 K and over a narrow stoichiometric range. Agreement with experiment was mixed, as the stability region for ternary C15 was in excellent agreement, whereas the stability region for the C14 phase was in poor agreement, possibly because the dynamic instability of UCo_2_ in the C14 phase or entropic factors not considered by this simple linear interpolation. Further research is needed to elucidate ternary phase stability.

## Conflicts of interest

There are no conflicts to declare.

## Supplementary Material

RA-013-D3RA00498H-s001
