# Supplementary material for: Investigating the role of phonons in the phase stability of uranium-based Laves phases
Source: RSC Adv. 2023 Mar 15;13(13):8646–56. doi: 10.1039/d3ra00498h (PMC10016338; doi:10.1039/d3ra00498h)
Supplement: RA-013-D3RA00498H-s001 [file RA-013-D3RA00498H-s001.pdf]

Electronic Supplementary Material (ESI) for RSC Advances.
This journal is © The Royal Society of Chemistry 2023

Supporting Information:

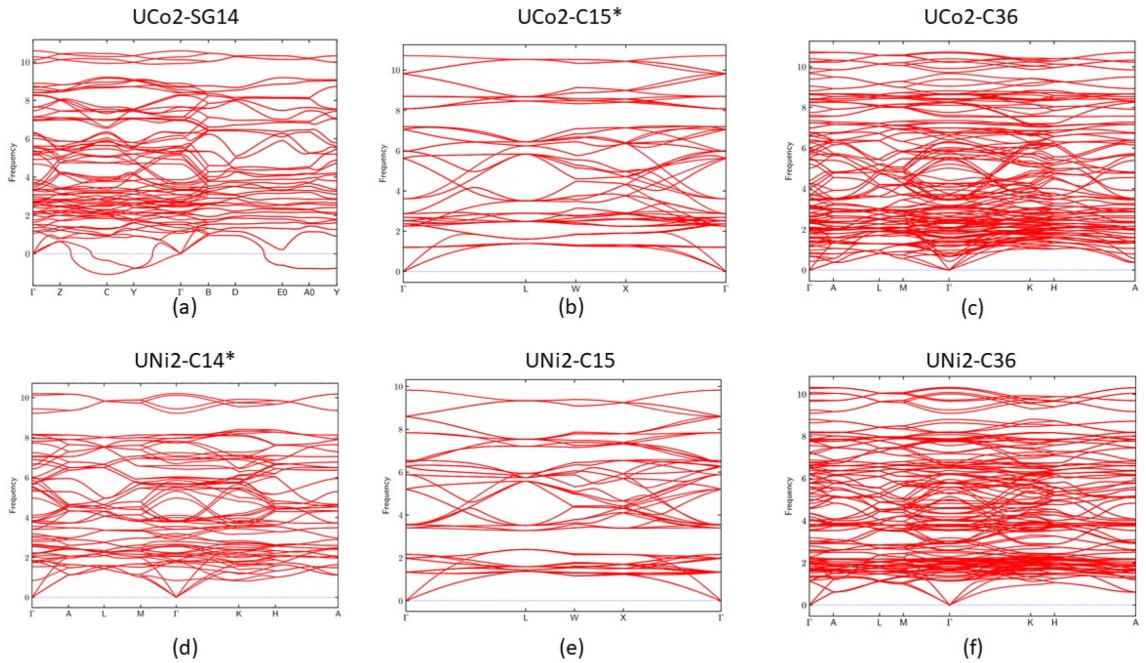

Supporting Fig. 1. Phonon band structures for all relaxed phases in THz. Note the extremely flat phonon modes in the C15 structures near 1 THz.

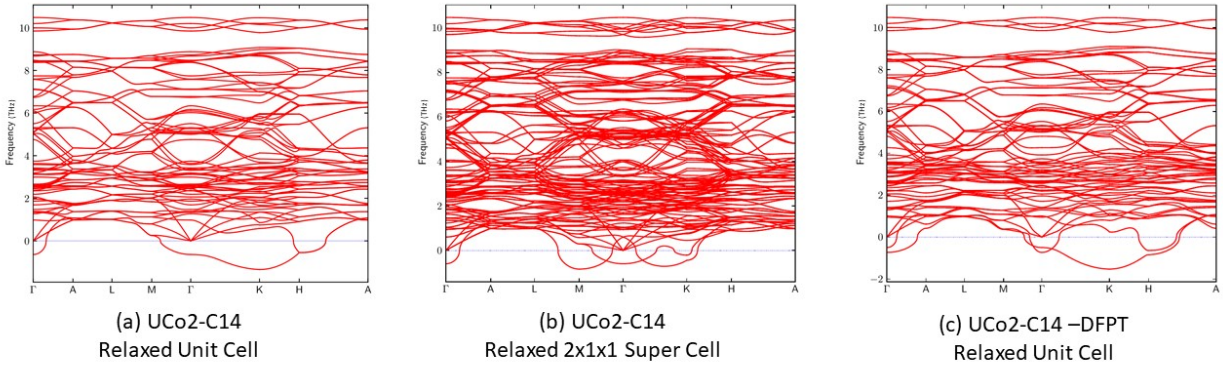

Supporting Fig. 2. Phonon band structures for relaxed UCo_2-C14 (SG176) calculated with different methods: (a) finite displacement method of the the relaxed unit cell, (b) finite displacement method on a relaxed 2 × 1 × 1 orthorhombic super cell, and (c) density functional perturbation theory on a relaxed unit cell. All of the methods predict an unstable phonon mode at the gamma point with an imaginary vibrational frequency.

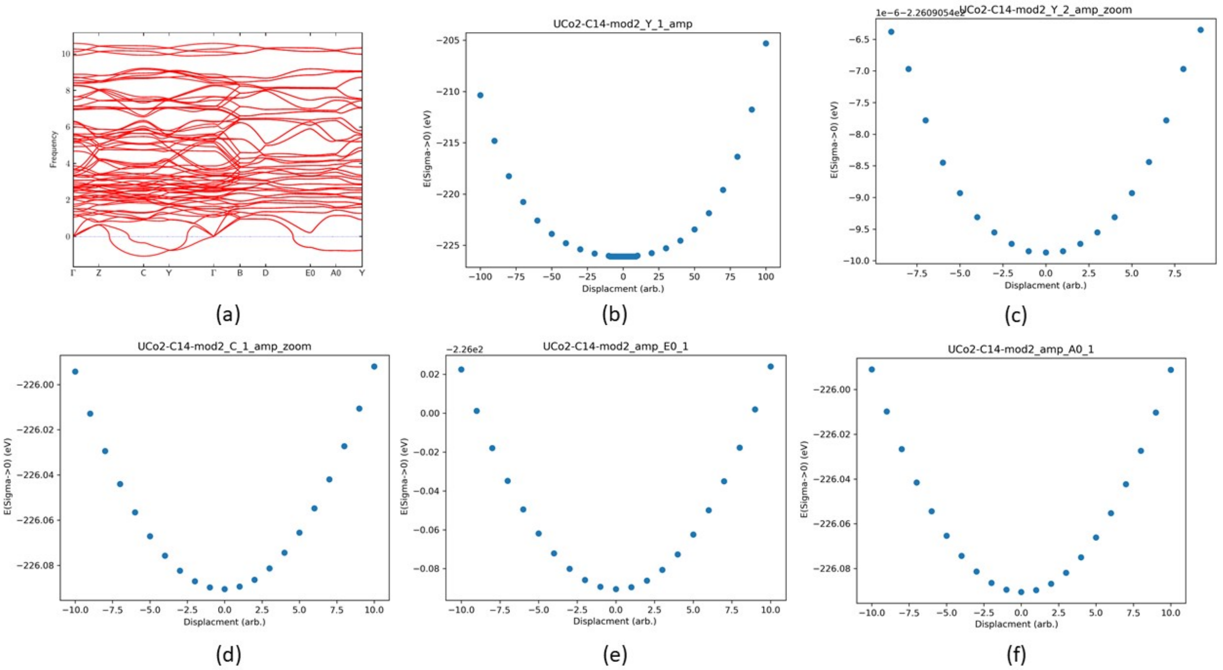

Supporting Fig. 3: (a) the phonon band structure of relaxed UCo2-SG14. (b-c) the energy surface associated with displacement of the atoms according to the vibrational eigenvectors at special k-points. Note that all of the energy surfaces are concave and do not indicate an obvious method of distortion to remove the unstable modes. It is possible the imaginary frequencies are computational artifacts.
